# Biovar-related differences apparent in the flea foregut colonization phenotype of distinct *Yersinia pestis* strains do not impact transmission efficiency

**DOI:** 10.1186/s13071-020-04207-x

**Published:** 2020-07-01

**Authors:** Athena Lemon, Janelle Sagawa, Kameron Gravelle, Viveka Vadyvaloo

**Affiliations:** Paul G Allen School for Global Animal Health, Washington State University, Washington, 99164 USA

**Keywords:** *Yersinia pestis*, Biovar, *Xenopsylla cheopis*, Glycerol, Transmission efficiency

## Abstract

**Background:**

*Yersinia pestis* is the flea-transmitted etiological agent of bubonic plague. Sylvatic plague consists of complex tripartite interactions between diverse flea and wild rodent species, and pathogen strains. Transmission by flea bite occurs primarily by the *Y. pestis* biofilm-mediated foregut blockage and regurgitation mechanism, which has been largely detailed by studies in the model interaction between *Y. pestis* KIM6+ and *Xenopsylla cheopis*. Here, we test if pathogen-specific traits influence this interaction by determining the dynamics of foregut blockage development in *X. cheopis* fleas among extant avirulent pCD1-*Y. pestis* strains, KIM6+ and CO92, belonging to distinct biovars, and a non-circulating mutant CO92 strain (CO92gly), restored for glycerol fermentation; a key biochemical difference between the two biovars.

**Methods:**

Separate flea cohorts infected with distinct strains were evaluated for (i) blockage development, bacterial burdens and flea foregut blockage pathology, and (ii) for the number of bacteria transmitted by regurgitation during membrane feeding. Strain burdens per flea was determined for fleas co-infected with CO92 and KIM6+ strains at a ratio of 1:1.

**Results:**

Strains KIM6+ and CO92 developed foregut blockage at similar rates and peak temporal incidences, but the CO92gly strain showed significantly greater blockage rates that peak earlier post-infection. The KIM6+ strain, however, exhibited a distinctive foregut pathology wherein bacterial colonization extended the length of the esophagus up to the feeding mouthparts in ~65% of blocked fleas; in contrast to 32% and 26%, respectively, in fleas blocked with CO92 and CO92gly. The proximity of KIM6+ to the flea mouthparts in blocked fleas did not result in higher regurgitative transmission efficiencies as all strains transmitted variable numbers of *Y. pestis*, albeit slightly lower for CO92gly. During competitive co-infection, strains KIM6+ and CO92 were equally fit maintaining equivalent infection proportions in fleas over time.

**Conclusions:**

We demonstrate that disparate foregut blockage pathologies exhibited by distinct extant *Y. pestis* strain genotypes do not influence transmission efficiency from *X. cheopis* fleas. In fact, distinct extant *Y. pestis* genotypes maintain equivalently effective blockage and transmission efficiencies which is likely advantageous to maintaining continued successful plague spread and establishment of new plague foci.
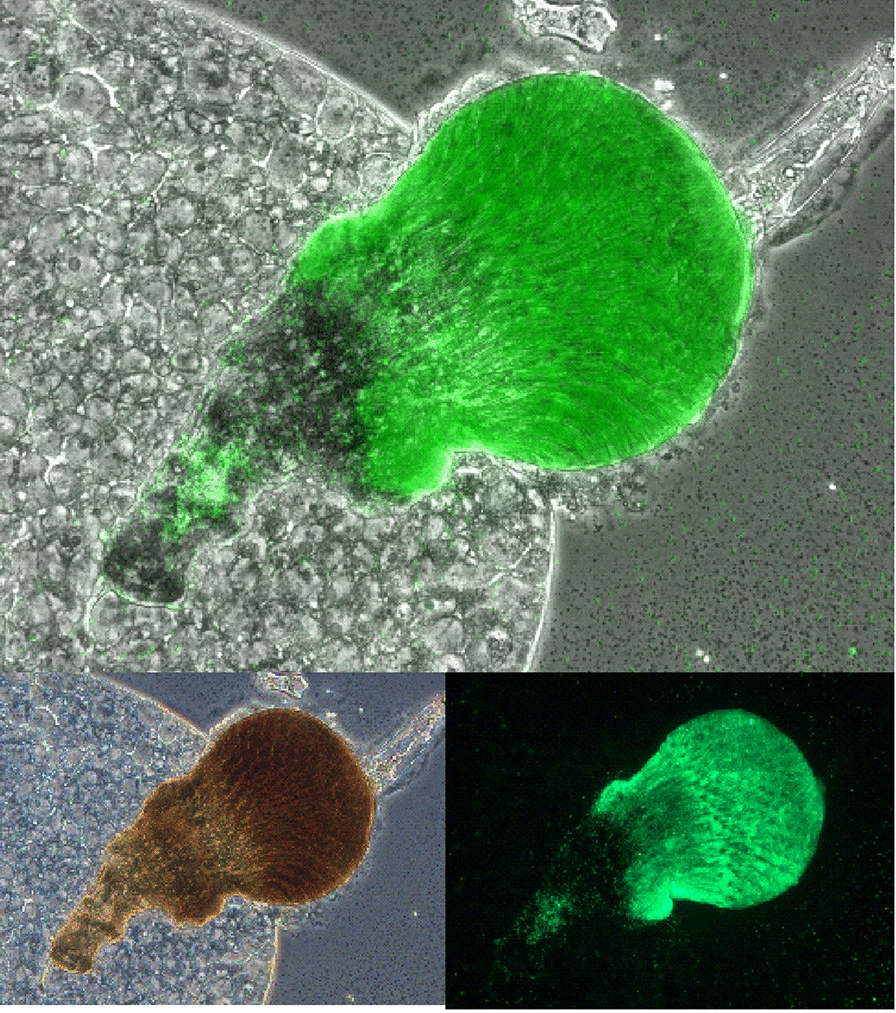

## Background

*Yersinia pestis*, the etiologic agent of bubonic plague, can be transmitted by a flea bite. In this model, the flea siphons blood into the midgut using muscles in its head that act as a peristaltic pump during feeding. The blood is drawn through the foregut which comprises the esophagus and proventriculus (PV), a valvular organ that ensures forward blood passage into the midgut for digestion while simultaneously preventing backflow [1]. However, when the flea acquires an infectious *Y. pestis* blood meal, the bacteria forms a biofilm in the PV that partially or completely obstructs function of this organ and blood passage to the midgut. This is referred to as biofilm-mediated blockage of the flea foregut [2]. During blockage, active ingestion of a subsequent blood meal causes distention of the esophagus and a localized build-up of hydrodynamic pressure in the proximal region of the esophagus immediately preceding the blocked PV [3]. An elastic recoil of the blood-filled esophagus follows, resulting in regurgitation of some *Y. pestis* biofilm back into the bite site thereby facilitating transmission. High blockage rates are thus synonymous with effective transmission, and both partial and complete blockage can cause blockage-mediated regurgitative transmission of *Y. pestis* [2, 4].

The biofilm-mediated blockage localized to the PV is comprised of dense multicellular aggregates of *Y. pestis* that are characterized by a self-produced extracellular polymeric substance (EPS). The EPS is primarily composed of poly-N-acetyl-d-glucosamine (PNAG) units [5]. The biosynthetic pathway of PNAG is encoded by the *hmsHFRS* locus which is positively post-transcriptionally regulated by the second messenger molecule, c-di-GMP. The two functional diguanylate cyclase (DGC) enzymes HmsD and HmsT synthesize c-di-GMP. HmsD predominantly affects biofilm production during flea colonization, whereas HmsT plays a lesser role in biofilm formation in the flea environment [6–8].

Historically, *Y. pestis* has been categorized into three clinically relevant biovars (bvs) differentiated by the biochemical abilities to ferment glycerol and arabinose, and reduce nitrate [9]: Antiqua (glycerol positive, arabinose positive and nitrate positive); Medievalis (glycerol positive, arabinose positive and nitrate negative); and Orientalis (glycerol negative, arabinose positive and nitrate positive). Recent phylogenetic analyses provide enhanced resolution of *Y. pestis* strain characteristics, evolutionary history of biovars and associated lineages, strain geographical pattern distinctiveness, and plague spread [10, 11]. Additionally, mathematical modelling reveals that branch 1 Orientalis and branch 2 Medievalis lineage strains spread significantly faster than others [12–15].

In laboratory based experimental studies, the prototype model of the interaction between *Y. pestis* strain KIM6+ and *Xenopsylla cheopis* has forged an understanding of the details regarding *Y. pestis* gut colonization, blockage development, transmission, and the bacterial genetic determinants for this process, including roles of *hmsHFRS*, *hmsT*, *hmsD* and *hmsP* genes [6, 7, 16]. The KIM6+ strain is a Medievalis bv branch 2 strain that was isolated from a Kurdish Iranian man [17, 18]. The oriental rat flea, *X. cheopis*, is implicated in past plague pandemics and current plague outbreaks in Madagascar [19] and is reputed to be an efficient vector of *Y. pestis* [20–22].

There remains minimal information regarding how different *Y. pestis* strains influence vector transmission efficiency. Genetic differences in pathogen strains and pathogen fitness are demonstrated determinants for vector transmission efficiency in animal and plant diseases as exemplified in the interactions between Zika virus and *Aedes aegypti*, bluetongue virus and *Culicoides sonorensis* midges, and *Xylella fastidiosa* and sharpshooter leafhoppers [23–25].

Similarly, to understand if strain-specific *Y. pestis* characteristics influence flea transmission efficiency, we comparatively characterized flea infection dynamics using two different *Y. pestis* strains in *X. cheopis* fleas, KIM6+ for which flea infection dynamics are well characterized and CO92. Use of the CO92 strain was motivated by several factors. First, CO92 belongs to the Orientalis branch 1 lineage comprising globally circulating strains, some directly implicated in causing the highest contemporary plague global case burden [12, 26, 27]. In contrast, Antiqua and Medievalis bvs are typically associated with enzootic rodent species in long term plague foci in Africa and Asia [28]. Secondly, CO92 was originally isolated from a fatal human case of pneumonic plague [29] and is primarily used for experimental infections in a murine plague model [30–32]. Thirdly, the most discerning genetic difference between CO92 and KIM6+ is that CO92 is incapable of metabolizing glycerol, allowing testing of a distinguishing metabolic trait in vector competency. Finally, CO92 produces significantly more biofilm than KIM6+ *in vitro* suggesting that it may have higher blockage rates in fleas [6]. Given these factors, we posited that CO92 may have enhanced abilities to colonize fleas and be transmitted from these insects.

## Methods

### Bacterial culture conditions

*Yersinia pestis* strains were cultured in heart infusion broth (HIB; BD Difco, New Jersey, USA) at 26 °C with aeration or grown on heart infusion agar (HIA). When appropriate, media were supplemented with carbenicillin (100 μg/ml), kanamycin (50 μg/ml) or trimethoprim (25 μg/ml). All strains, plasmids and oligonucleotides are listed in Tables 1 and 2.

**Table 1:** List of strains and plasmids used

*Yersinia pestis* strains and plasmids used	Characteristics	Reference
CO92	Pgm*+* pCD1- pMT1+ pPCP1+, parental strain	[32]
CO92 *glmS-pstS::P*_*trc*_*GFP*	CO92 with chromosomally inserted *gfp* for imaging	This study
CO92 *glpD+*	Defective *glpD′* allele replaced with functional *glpD* gene from KIM6+ in the chromosome	[32]
CO92 *glpD+glmS-pstS::P*_*trc*_*GFP*	defective *glpD′* allele replaced with functional *glpD* gene in the chromosome, with chromosomally inserted GFP for imaging	This study
CO92gly	Defective copy of the *glpD′* allele replaced with functional *glpD* in the chromosome, and carries *glpFKX* from KIM6+ on pTpGLPFKX6	This study
CO92gly *glmS-pstS::P*_*trc*_*GFP*	CO92gly with chromosomally inserted *gfp* for imaging	This study
CO92 Δ*hmsD*::*frt*	Δ*hmsD*	This study
CO92 Δ*hmsT*::*frt*	Δ*hmsT*	This study
KIM6+	Pgm*+* pCD1- pMT1+ pPCP1+, parental strain	[16]
KIM6+::*kan*^*R*^	KIM6+ with chromosomally inserted kanamycin resistance for co-infections	[39]
KIM6+ pAcGFP1	KIM6+ with GFP for imaging	This study
pAcGFP1	Carries AcGFP1, Cb^r^	Clontech (Mountain View, CA, USA)
pTNS2	TnsABC+D specific transposition pathway, Cb^r^	[33]
pKD13	Source of kanamycin resistant cassette and flanking Frt sequence	[35]
pFLP3	Recombinase, Cb^r^, Tet^r^	[33]
pUC18R6KT-mini-Tn7T-Km	Cloning vector for Tn7 insertion, Cb^r^, Km^r^	[33]
pGP-Tn7-P_trc_*gfpmut3*	Source of P_trc_*gfpmut3*, Cb^r^	[34]
pUC18R6KT-mini-Tn7T-P_trc_*gfpmut3*	Cloning vector for Tn7 insertion of P_trc_*gfpmut3*, Cb^r^, Km^r^	This study

*Abbreviations*: Cb^r^, carbenicillin resistance; Km^r^, kanamycin resistance; Tet^r^, tetracycline

**Table 2:** List of oligonucleotides used

Oligonucleotide	Reference	Purpose	Sequence
p130	[35]	Amplify kanamycin cassette and check insertion	GTGTAGGCTGGAGCTGCTTC
p131	[35]	Amplify kanamycin cassette and check insertion	ATTCCGGGGATCCGTCGACC
p407/pGPTn7pro	This study	To clone *gfpmut3* into pUC18R6KT-mini-Tn7T-Km	ATAGGAATTCCTTCTCGAGCGACTGCACGGTGC
p408/pGFPTn7R	This study	To clone *gfpmut3* into pUC18R6KT-mini-Tn7T-Km	CAGAGCGCTTTTGAAGCTAATTCGATC
p544	[51]	To mutate *hmsT*	ACGTGGTACAACATGCTGACGGTTG
p545	[51]	To mutate *hmsT*	GAAGCAGCTCCAGCCTACACCATAATATCGTGCTGTCAGTAGACTAATAAATC
p546	[51]	To mutate *hmsT*	GGTCGACGGATCCCCGGAATGATTAACTCACTGAACATACGGACGCTCTATG
p547	[51]	To mutate *hmsT*	CTTCTCATCATCATCTGATGCGGCC
p548	[51]	To mutate *hmsD*	GCCAGTAATATCAATGCAGATATTCTGCG
p549	[51]	To mutate *hmsD*	GAAGCAGCTCCAGCCTACACCATAGATTGGTTTTTTATCGTGTTCATCGT
p550	[51]	To mutate *hmsD*	GGTCGACGGATCCCCGGAATGTGACATGCAGAAAATGCAAAACAAGCGTT
p551	[51]	To mutate *hmsD*	CGTTAGGACGCGGTGATAATAATGGCG
p556	This study	To verify *hmsD* mutation	AGCGCAGAATTCGTACCTAACGCCGATTCAACC
p577	This study	To verify *hmsD* mutation	ATCCACTTCATCCTGGCAGC
p578	This study	To verify *hmsT* mutation	CGCAAGACTCTCGCTTCTTTG
p579	This study	To verify *hmsT* mutation	TTCCACGTTTGTAGCAAACCC

### GFP insertion into *Y. pestis*

*Yersinia pestis* strains were chromosomally tagged at the *attTn7* site with *gfpmut3* using a Tn7-based system [33]. To do this, *gfpmut3* under the control of the P_trc_ promoter was restricted from pGP-Tn7-P_trc_*gfpmut3* [34] and cloned into matching restriction sites of pUC18R6KT-mini-Tn7T-Km [33] to create pUC18R6KT-mini-Tn7T-P_trc_*gfpmut3* and electroporated into electrocompetent strains of *Y. pestis*. These plasmid constructs were verified by sequencing. Alternatively, *Y. pestis* electrocompetent strains were transformed with pAcGFP1 and selected on HIA containing carbenicillin.

### Deletion of *hmsD* and *hmsT*

Deletion of *hmsD* and *hmsT* was achieved by homologous recombination using the lambda red recombinase system [35]. Flanking regions of approximately 500 bp upstream and downstream of the *hmsD* and *hmsT* genes were first amplified by PCR. The resulting PCR products were gel-purified and combined using splice-overlap extension (SOE) PCR with a kanamycin resistance cassette flanked by *frt* sites that were previously amplified from the plasmid pKD13 [35]. Electrocompetent *Y. pestis* CO92 carrying pKOBEG and expressing the recombinase was transformed with purified SOE PCR fragments [36]. Recombinant strains were picked on kanamycin and confirmed by PCR. The kanamycin resistance cassette introduced in the previous step was resolved by the introduction of pFLP3. Deletion mutants CO92 Δ*hmsD*::*frt* and CO92 Δ*hmsT*::*frt* were confirmed by sequencing.

### Flea infections

*Yersinia pestis* strains confirmed as Pgm-positive were cultured overnight in heart infusion broth (HIB) as described previously [37]. Bacteria were added to 6 ml of sodium heparinized mouse blood (BioIVT, New York, USA) at a concentration of ~1 × 10^9^ CFU/ml [38]. *Xenopsylla cheopis* fleas were then allowed to feed on the infected blood using a previously described artificial feeding apparatus [16, 39]. Only fleas that took full blood meals were selected. Fifty male and 50 female fleas were maintained at 21 °C and 75% relative humidity, fed twice weekly on uninfected mice and monitored for proventricular blockage over a period of 28 days as previously described [16]. To perform co-infections, the protocol was followed as previously described [39].

### Determining *Y. pestis* CFU in the infectious blood meal and infected flea samples

After artificial infection, a sample of the infected blood meal was serially diluted and plated on Columbia blood agar (Hardy Diagnostics, California, USA) to determine *Y. pestis* CFU/ml in the blood. To quantify infection rate and bacterial load of the fleas, 20 infected fleas were collected immediately after the infectious blood meal, and at days 7, 14 and 28 post-infection. Fleas were subjected to individual plating on brain heart infusion (BHI) agar (BD Difco) supplemented with 1 μg/ml irgasan and 10 μg/ml hemin to determine CFU count. To determine CFU/flea from co-infected fleas, samples were treated as previously described, but plated on heart infusion agar with and without 50 μg/ml kanamycin to select for KIM6+::*kan*^*R*^ on individual sets of plates respectively [39].

### Imaging and esophageal scoring of fleas

Blocked fleas were dissected in PBS to isolate the flea gut. A 1.5 mm glass cover slip was then placed over the samples for imaging. Flea guts were imaged with a Leica DMi8 epifluorescence microscope (Leica Microsystems, Illinois, USA) with phase contrast and a GFP filter cube (Ex. 470/40nm, Em. 525/50nm). Esophageal images were first evaluated for valid scoring by determining if: (i) the esophagus was intact; and (ii) the entire esophagus was visible. If these criteria were met, the esophagus was scored for presence of GFP in which presence of GFP in < 1/3 the length of the esophagus was assigned a proximal colonization status, and presence of GFP in ≥ 1/3 the length of the esophagus was assigned a distal-medial colonization status.

### Mass transmission experiment

For transmission experiments, fleas that took an infectious blood meal were housed as described above. On days 7 and 14, the fleas involved in the transmission experiment fed on 5 ml of sterile sodium heparinized CD-1 (ICR) mouse blood (BioIVT) using the artificial feeding system. After 60 min, fleas were examined microscopically to determine how many had taken a blood meal, and of those, how many were blocked. Transmission experiments were performed as previously described [40]. Immediately after each transmission feeding, all 5 ml of blood were removed and plated on Columbia blood agar plates. The interior of the feeder was rinsed 24 times with 1 ml of PBS and these washes were pooled. Pooled washes were centrifuged at ~12,000× *g* for 10 min, supernatant removed, and the pellet was plated onto Columbia blood agar plates. The external surface of the mouse skin was disinfected with 70% ethanol twice to reduce surface contaminants and cut into small pieces that were homogenized in PBS in a FastPrep homogenizer (MP Biomedicals, California, USA) to release any bacteria deposited by flea bite into the mouse skin. Skin sample supernatants were pooled and treated in the same manner as the pooled washes. After incubation at 28 °C for 48 h, *Y. pestis* colonies were counted and confirmed by replicate patch plating on both Yersinia selective agar plates and Congo Red agar. At each time point post-infection 20 fleas were collected for enumeration of bacterial loads. To maintain the normal bi-weekly schedule of feeding, fleas were also fed on neonatal mice on days 4 and 11.

### Statistical analysis

All analyses were performed using GraphPad Prism 7 (GraphPad Software Inc., La Jolla, California, USA.). Fisher’s exact test was performed with the fisher.multcomp package in R (3.6.1). The statistical tests that were used and relevant *P*-values are indicated in the figure legends and results.

## Results

### KIM6+ and CO92 infected *X. cheopis* fleas have similar blockage rates

**Fig. 1:**
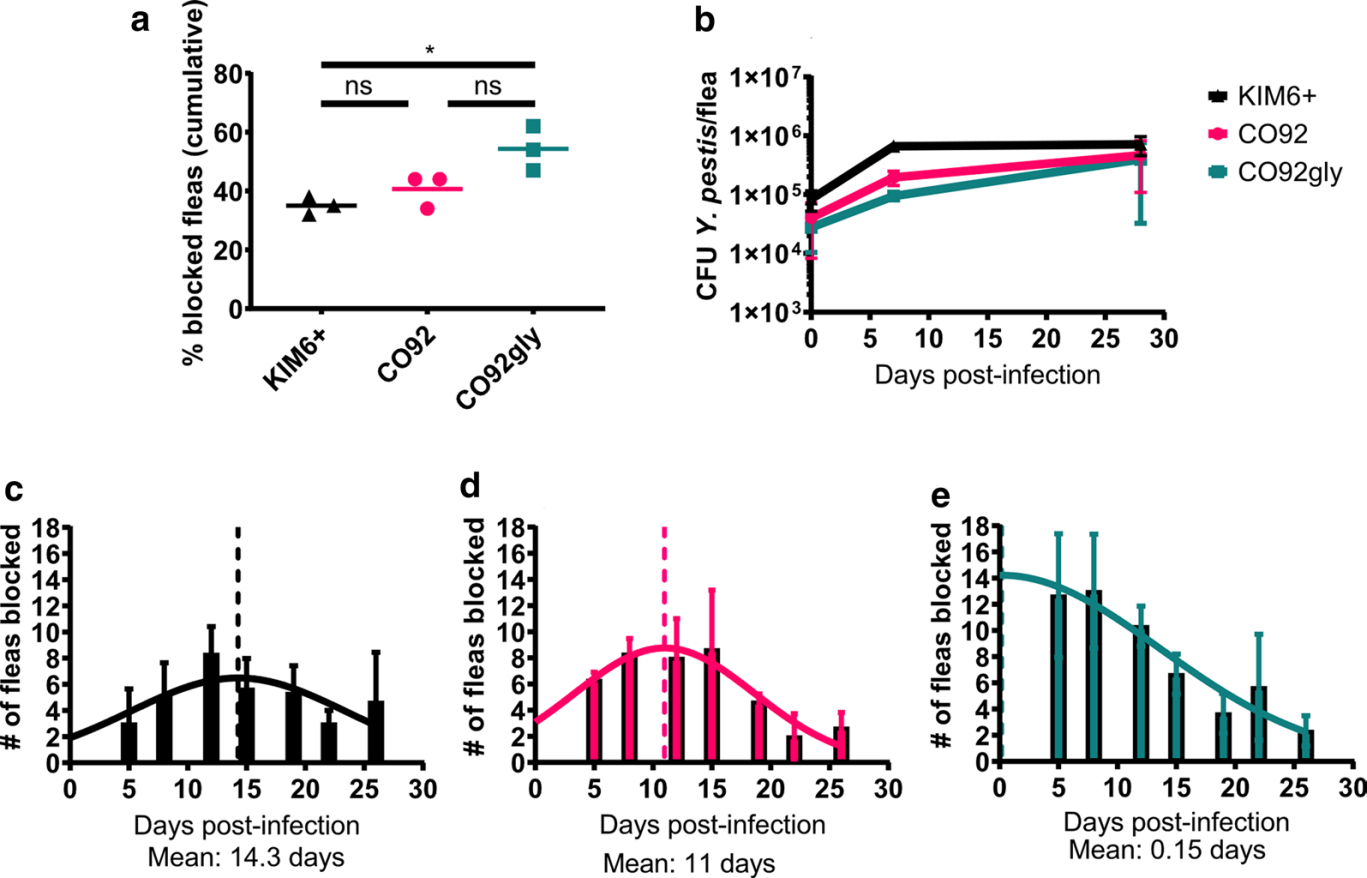
**a** Foregut blockage and infection dynamics in fleas infected with CO92, KIM6+ and CO92gly. Cumulative flea blockage rate of strains KIM6+ (black), CO92 (pink) and CO92gly (teal). Error bars represent the mean of three independent flea infection experiments per strain. *P ≤ 0.05. **b** Growth kinetics of strains KIM6+, CO92, and CO92gly during infection of *X. cheopis* fleas, error bars represent the mean ± SD of three independent flea infections per strain with each time point reflecting 15–20 fleas per strain per independent experiment. Histograms representing temporal incidence of blockage in KIM6+ (**c**), CO92 (**d**), and CO92gly (**e**) were fit to a Gaussian model, and the dashed line indicates mean time to peak blockage. CO92gly had an earlier mean to peak blockage than KIM6+ or CO92

To determine if *Y. pestis* strain specific distinctions can be made in the interactions of *Y. pestis* and its prototype flea vector, *X. cheopis*, blockage rates and flea colonization dynamics were directly compared between CO92 and KIM6+ strains. The distinctive phenotypic difference between KIM6+ and CO92 is the inability of CO92 to metabolize glycerol. Therefore, to simultaneously determine the role of glycerol utilization in potential differences that may be uncovered in flea infection dynamics between CO92 and KIM6+, we also tested a previously reported CO92 strain [32] that was restored in ability to metabolize glycerol (referred to as CO92gly). For this, *X. cheopis* fleas were artificially infected with CO92, KIM6+ or CO92gly strains to determine blockage rate, flea infection rate, and bacterial burden per flea over time. We found that the cumulative blockage rate of fleas infected with KIM6+ or CO92 over the period of a month averaged approximately 37% (SD ± 3.00%), and 40% (SD ± 5.78%) respectively (Fig. 1a). This was consistent with previous reports of blockage rates between 25–45% for the KIM6+ strain [40]. Strikingly, mean blockage rate of CO92gly infected fleas was 54% (SD ± 7.5%), significantly higher (t-test: *t*_(4)_= 4.143, *P *= 0.0143) than blockage rates for KIM6+ (Fig. 1a). However, no significant differences in bacterial burden per flea and rate of flea infection were noted for all three strains (Fig. 1b).

A detailed assessment of temporal incidence of blockage demonstrated that peak incidence of blockage occurred at 14.3 days post-infection for KIM6+ (Fig. 1c), but at 11 days post-infection for CO92 (Fig. 1d). In contrast, temporal incidence of blockage did not follow a normal distribution and was predicted to peak at a physiologically unrealistic 0.15 days (~3 h post-infection) for CO92gly (Fig. 1e).

### KIM6+ biofilm blockage extends the full length of the *X. cheopis* esophagus

**Fig. 2:**
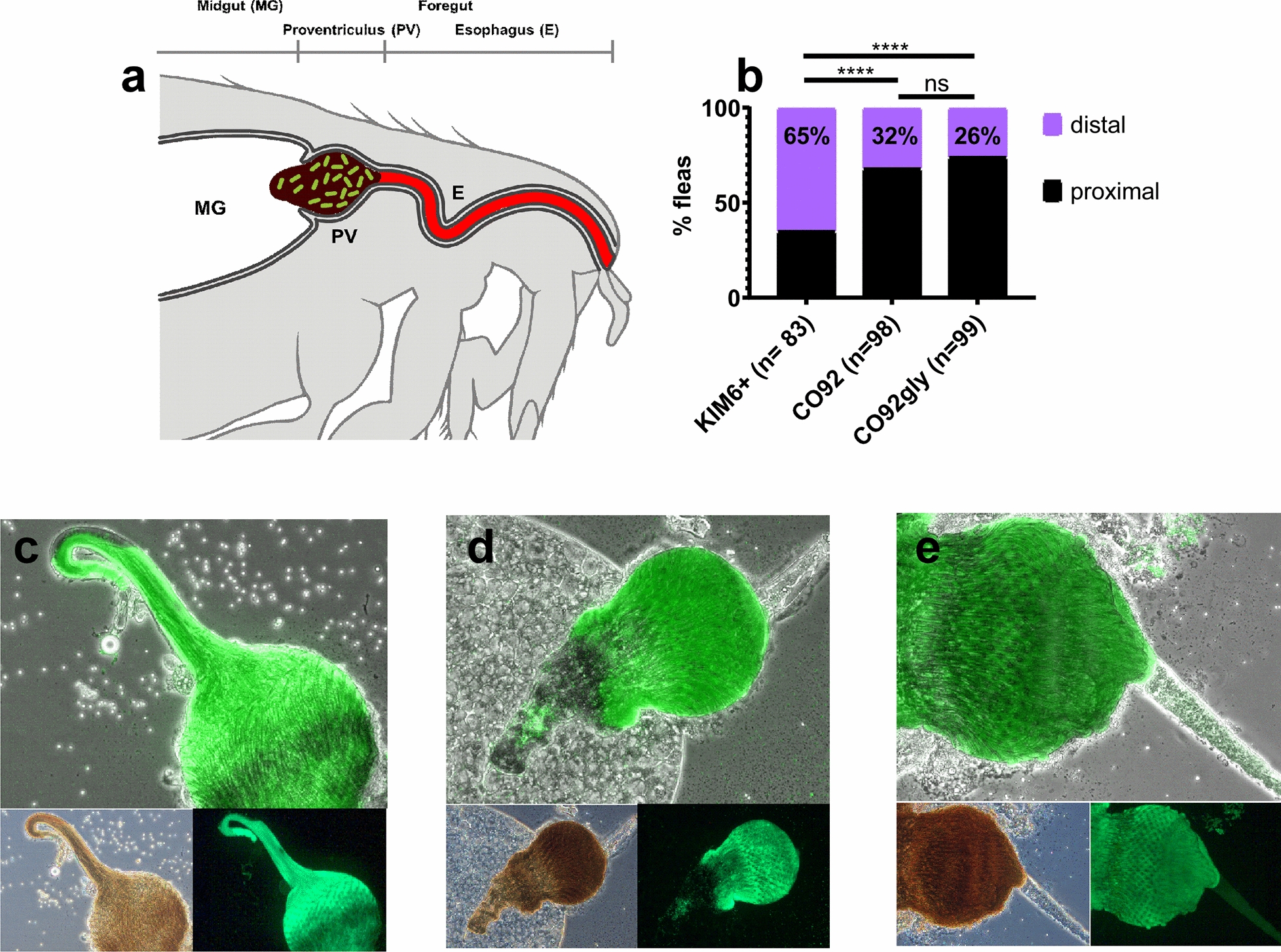
**a** KIM6+ colonizes the entire length of the esophagus in blocked fleas. *Xenopsylla cheopis* fleas infected with *Y. pestis* strains form a biofilm blockage in the foregut proventriculus (PV) that may extend through the esophagus (E), preventing a blood meal from reaching the midgut (MG), and resulting in regurgitative transmission. **b** Midguts of fleas blocked with GFP-labelled *Y. pestis* strains were dissected and imaged to determine localization of bacterial biofilm in the flea foregut. Bacterial localization that extended the entire length of the esophagus was scored as distal esophagus localization and one-third to no colonization of the esophagus was scored as proximal esophagus colonization. Significant differences in percentage esophagus colonization was determined by Fisher’s exact test. Representative images of dissected flea midgut showing PV and esophagus are shown for KIM6+ (**c**), CO92 (**d**), and CO92gly (**e**). ****P ≤ 0.0001. *Abbreviation*: ns, not significant

No quantitative differences in flea blockage rate, mortality, or infection were noted between the KIM6+ and CO92 strains. However, in preliminary studies we noted that KIM6+ colonized the entire length of the flea esophagus, as well as the PV, whereas CO92 primarily colonized the PV alone. Microscopic visualization prior to application of a coverslip eliminated any concerns that the phenotype observed resulted from the process of the coverslip laying down on the gut. To quantitatively assess this phenotype, we developed an esophageal pathology scoring rubric which enabled parsing of colonization status into major (distal-medial) and minor (proximal) with respect to *Y. pestis* esophagus colonization of the flea foregut. Intriguingly and consistent with preliminary observations, a significantly greater (Fisher’s exact test: OR: 0.2534, 95% CI: 0.1164–0.5457, *P * < 0.0001) 65% of KIM6+ blocked fleas compared to only 32% of fleas blocked with CO92 colonized most of the surface of the flea esophagus (Fig. 2a–e). The CO92gly strain maintained a similar foregut colonization phenotype to the parental strain (Fisher’s exact test: OR: 1.339, 95% CI: 0.6113–3.069, *P* = 0.4360).

### Transmission of KIM6+, CO92 and CO92gly is not significantly different

**Fig. 3:**
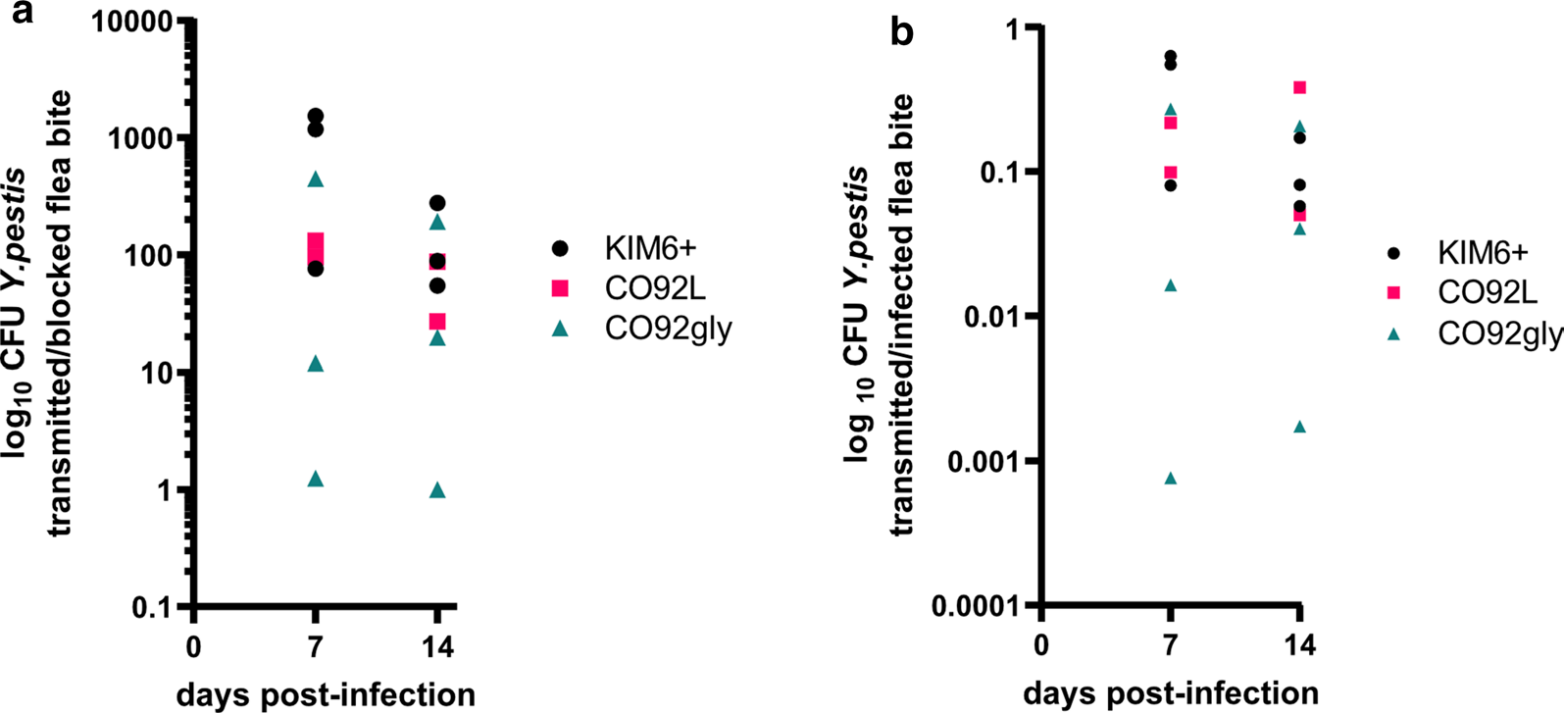
KIM6+, CO92, and CO92gly are transmitted from infected fleas at similar efficiencies. *Yersinia pestis* CFU transmitted by *X. cheopis* infected with KIM6+ (black triangles), CO92 (pink circles), and CO92gly (teal squares) during *in vitro* mass transmission experiments, per blocked flea bite (total number of transmitted CFUs/ number of blocked fleas) (**a**) and per infected flea bite (total number of transmitted CFUs/[number of fed fleas × % infected fleas]) (**b**). Fleas were fed on mouse blood seeded with ~10^9^*Y. pestis* CFU/ml and were subjected to mass artificial transmission experiments 7 and 14 days after a single infectious blood meal. Symbols represent three independent experiments for KIM6+ and CO92gly, and two independent experiments for CO92. No significant difference in CFU *Y. pestis* transmitted per blocked flea or per infected flea bite between strains was noted using one-way ANOVA analysis

Full esophagus colonization by the sticky *Y. pestis* biofilm reflects that bacteria are in closer proximity to the feeding mouthparts and can likely be more efficiently regurgitated back into the flea bite site. Alternatively, the impediment caused by full *Y. pestis* esophageal colonization may reduce build-up of the hydrodynamic forces and pressure in the esophagus, resulting in fewer *Y. pestis* cells being dislodged and regurgitated from the biofilm blockage. To address this, we asked if the esophageal colonization differences observed among the CO92, CO92gly and KIM6+ strains might impact regurgitative transmission of bacteria into the flea bite site. Cohorts of *X. cheopis* fleas were infected with the *Y. pestis* strains of interest. Once a week for two weeks, mass-transmission experiments were conducted to determine the total CFU of each *Y. pestis* strain that is regurgitated by infected fleas into an artificial blood-feeding apparatus containing sterile blood. For all the strains tested, KIM6+, CO92 and CO92gly, a wide and variable range of *Y. pestis* CFU was transmitted per feeding (Fig. 3, Table 3). Although not statistically significant, fewer CO92gly CFUs were transmitted per blocked flea bite with similar numbers of blocked fleas occurring in all three strains (Fig. 3a, Table 3). Similarly, fewer CO92gly CFUs were transmitted per infected flea bite (Fig. 3b).

**Table 3:** *Xenopsylla cheopis* mass transmission summary

Days PI	No. of fleas fed	% infected	CFU/infected flea	No. of fleas blocked	CFU transmitted
*X. cheopis* infected with KIM6+ mass transmission summary					
Experiment 1 (1.10 × 10^9^ CFU/ml blood)					
0	–	80	1.20E+05	–	–
7	80	95	6.85E+05	8	607
14	88	100	7.81E+05	13	710
Experiment 2 (2.46 ×10^9^ CFU/ml blood)					
0	–	100	2.19E+05	–	–
7	206	95	6.35E+05	11	10,749
14	120	95	4.50E+05	7	1942
Experiment 3 (6.70 × 10^8^ CFU/ml blood)					
0	–	100	1.08E+05	–	–
7	206	100	2.82E+05	7	12,957
14	120	90	2.69E+05	7	620
*X. cheopis* infected with CO92 mass transmission summary					
Experiment 1 (1.03 × 10^9^ CFU/ml blood)					
0	–	100	2.76E+03	–	–
7	132	90	1.11E+05	27	2583
14	65	95	3.46E+05	27	2353
Experiment 2 (2.27 × 10^9^ CFU/ml blood)					
0	–	100	8.87E+04	–	–
7	183	95	2.62E+05	13	1714
14	81	100	3.37E+05	15	405
*X. cheopis* infected with CO92gly mass transmission summary					
Experiment 1 (2.06 × 10^9^ CFU/ml blood)					
0	–	95	2.51E+04	–	–
7	103	85	4.04E+05	12	143
14	52	85	1.81E+05	9	177
Experiment 2 (1.45 × 10^9^ CFU/ml blood)					
0	–	65	1.19E+04	–	–
7	92	90	7.14E+05	5	2233
14	54	75	8.94E+04	7	7
Experiment 3 (6.70 × 10^8^ CFU/ml blood)					
0	–	95	7.00E+04	–	–
7	88	75	7.32E+05	4	5
14	55	85	9.45E+05	5	961

*Abbreviation*: PI, post-infection

### KIM6+ and CO92 are equally fit during competitive flea co-infection

**Fig. 4:**
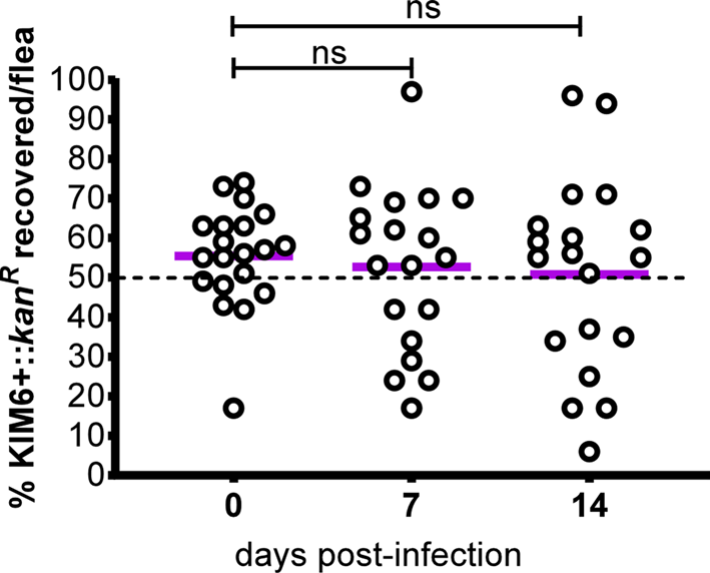
KIM6+ and CO92 are equally fit during competitive co-infection in fleas. *Xenopsylla cheopis* fleas were co-infected with a 1:1 ratio of CO92 and KIM6+::*kan*^*R*^. The *Y. pestis* bacterial loads were determined for 18–20 fleas at time points 0 (2 h post-acquisition of the co-infected blood meal), 7 and 14 days post-infection. The percentage of KIM6+::*kan*^*R*^ in the co-infection was determined and plotted for each time point. One of two independent experiments with similar outcomes is shown. A Student’s t-test was used to determine statistical significance between time points. *Abbreviation*: ns, not significant

The distinctive flea gut colonization phenotypes exhibited by the CO92 and KIM6+ strains may be important traits for flea infection that can only be discerned in more sensitive competitive fitness studies [37, 39]. To test this, we infected fleas with a 1:1 ratio of both strains. At 2 h post-infection, and by days 7 and 14 post-infection, there was no significant change in the ratio of each strain per flea. This data implies that KIM6+ and CO92 are equally fit during competitive co-infection in fleas (Fig. 4).

### CO92 *hmsT* and *hmsD* diguanylate cyclase encoding genes have matching roles in blockage formation to KIM6+ *hmsT* and *hmsD* genes

We additionally considered that the phenotypic differences in foregut colonization noted between KIM6+ and CO92 was a consequence of disparate nutritional sensing due to the biochemical differences in the strains. Localized environmental signals including nutrient signals in the flea foregut may contribute to differential regulation of the diguanylate cyclase encoding genes [41–43] and alterations in c-di-GMP signaling and biofilm production. We know that in the KIM6+ strain, the *hmsD* gene is essential for blockage while the absence of *hmsT* reduced blockage rates by approximately 50% [7]. However, the individual contributions of HmsD and HmsT diguanylate cyclases to blockage development in CO92 strains is unknown [7]. We therefore compared blockage rates between CO92 mutants in *hmsT* and *hmsD* alongside the isogenic parental CO92 strain and found an 82% and 39% reduction in blockage for mutants relative to the CO92 parental strain, respectively (Table 4). The roles of HmsT and HmsD in blockage formation by CO92, as such, recapitulated that occurring in the KIM6+ strain (Table 4).

**Table 4:** CO92 c-di-GMP diguanylate cyclases have similar contributing roles to blockage formation as their homologs in KIM6+

	*Y. pestis* strain					
	CO92	CO92 Δ*hmsD*::*frt*	CO92 Δ*hmsT*::*frt*	KIM6+^a^	KIM6+ Δ*hmsD*^a^	KIM6+ Δ*hmsT*^a^
Mean % reduction in mutant blockage *vs* parental strain	na	82	39	na	92	57
Mean CFU/flea (0 dpi)	3.1 × 10^4^	9.4 × 10^3^	2.3 × 10^4^	1.3 × 10^5^	5.1 × 10^4^	5.2 × 10^4^
Mean CFU/flea (28 dpi)	1.3 × 10^6^	7.9 × 10^5^	3.7 × 10^5^	5.4 × 10^5^	3.0 × 10^5^	4.7 × 10^5^

^a^All KIM6+ data derived from [7]

*Notes*: Data is reflective of one experiment, 20 fleas plated per time point

*Abbreviations*: na, not applicable; dpi, days post-infection

## Discussion

The present study aimed to understand the contribution of genetically and biochemically distinct *Y. pestis* strains to vector competence of the prototype flea vector species of plague, *X. cheopis* [37]. Our studies reveal that pathogen-specific differences in colonization of the flea foregut between the CO92 and KIM6+ strains do not result in differences in blockage rates, competitive fitness, *in vitro* transmission efficiency, or demonstrate different contributions of diguanylate cyclases to blockage during infection of *X. cheopis* fleas. The present blockage rate data of CO92 to KIM are consistent with previous results with two other Orientalis strains, 195/P (16) and GB [44].

In this study we additionally examined whether the ability to utilize glycerol, present in KIM6+ but absent in CO92, may contribute to vector competence. This was motivated by previous work demonstrating that genes encoding glycerol uptake are specifically induced in KIM6+ blocked fleas suggesting that active glycerol metabolism occurs in this strain during flea infection [37, 45]. Loss of glycerol uptake and utilization in CO92 is due to mutations in *glpD* and the *glpFKX* operon, but a previous study undertook restoration of this biochemical ability by complementing the CO92 strain with intact copies of *glpD* and the *glpFKX* genes [32]. Functionally restoring glycerol utilization to the CO92 wild-type strain did not impact virulence in a mouse model or biofilm production *in vitro*, suggesting that glycerol utilization is not essential for these functions [32].

Our study attributes re-establishment of glycerol utilization in the CO92gly strain to enhanced blockage rates but not changes in growth, flea bacterial burdens, or esophageal colonization in fleas. Principally, blockage is dependent on production of a biofilm EPS matrix that allows a robust adherence of bacterial aggregates to the PV a few days post-acquisition of the infected blood meal [46]. EPS production likely occurs at elevated levels in CO92gly leading to hyperbiofilm production in the flea gut. This may be counterproductive to transmission if the cohesive nature of the hyperbiofilm prevents easy dislodging of bacteria by hydrodynamic forces, resulting in lower numbers of regurgitated bacteria. Interestingly, CO92gly-infected fleas tended to transmit fewer bacteria despite a significantly higher blockage rate. The phosphodiesterase activity of *Y. pestis* HmsP is required to degrade c-di-GMP and decrease EPS production [6, 47] and an HmsP mutant of *Y. pestis* is a hyperbiofilm producer generating bacterial aggregates that are extraordinarily cohesive [47]. Alteration of c-di-GMP levels through regulation of its synthesis and degradation enzymes is suggested to occur through sensing of nutritional signals [37, 43], which, in this case could be conferred by the ability to ferment glycerol that is normally absent in CO92 strains. Glycerol utilization in the CO92 genetic background does not, however, appear to bode well for optimal transmission efficiency. First, the peak blockage incidence of the CO92gly strain three hours post-infection is physiologically improbable for EPS synthesis. Secondly, this improbable peak blockage incidence and blockage rate strays from the norm exhibited by the extant, circulating epidemic strains, CO92 and KIM6+. Thirdly, this strain shows a tendency to transmit fewer bacteria reflecting reduced transmission efficiency. Marked genome rearrangements also distinguish CO92 from KIM strains and may contribute to gene expression alterations relevant to metabolic processes in these strains [17]. Incorporation of an ability to metabolize glycerol within the existing CO92 metabolic networks may thus be deleterious to a metabolic physiology that supports optimal flea transmission competency. In KIM6+ glycerol utilization is likely metabolically innocuous. This may explain the loss of the pathway in Orientalis strains which have achieved global distribution.

One significant observation in this study is the predominant occurrence of extended colonization of the esophagus in KIM6+ blocked fleas. The physical anatomy of the flea foregut with regards to musculature surrounding the PV and the width of both the PV and esophagus have proven to be important determinants of flea vector transmission efficiency [40, 48]. The observation that the KIM6+ biofilm blockage ‘infiltrates’ the esophagus was made previously and described to be responsible for distending the esophagi width of *X. cheopis* and *Oropsylla montana* fleas [40]. The wider *O. montana* esophagus facilitates regurgitative transmission of greater numbers of bacteria in keeping with its greater width in these reports. However, our results did not reveal any difference in the CFU transmitted by KIM6+ and CO92 blocked *X. cheopis* fleas. In fact, this finding is consistent with previous studies that show similar variable ranges of bacteria being transmitted from *X. cheopis* fleas infected with KIM6+, and that transmission by flea bite is a relatively inefficient process requiring bolstering by high flea indices [38, 49]. We speculate that the relevance of esophageal colonization differences regarding regurgitative transmission may differ in other flea species with anatomical foregut characteristics that are distinct from that of *X. cheopis*. Certainly, the proposition that natural endemic foci harbor discrete co-evolved *Y. pestis* strain-flea vector species associations is a provocative one that may give credence to this notion [10, 50].

## Conclusions

Collectively, our study has highlighted that pathogen-specific traits occur during flea colonization, yet these differences cumulatively amount to comparable efficiencies in *X. cheopis* fleas to transmit both extant CO92 and KIM6+ strains. This is consistent with the idea that it is evolutionarily favorable for this pathogen to non-selectively be transmitted from a broad range of competent flea vectors. In the backdrop of highly complex plague ecology, further analyses are needed to have a broad understanding of the nuances in vector competence that supports natural maintenance of this pathogen in flea-rodent cycles worldwide.

## Data Availability

All data generated or analyzed during this study are included in this published article.

## Abbreviations

PV: proventriculus
EPS: extracellular polymeric substance
SD: standard deviation
PNAG: poly-N-acetyl-d-glucosamine
DGC: diguanylate cyclase
bvs: biovars
HIB: heart infusion broth
HIA: heart infusion agar
SOE: splice-overlap extension
CFU: colony forming units
GFP: green fluorescent protein
Ex: excitation
Em: emission
